# Emerging Infections Network Survey of Screening for Cryptococcal Antigenemia, United States, 2024

**DOI:** 10.3201/eid3107.250295

**Published:** 2025-07

**Authors:** Kaitlin Benedict, Alexander Jordan, Jeremy A.W. Gold, Dallas J. Smith, Tom Chiller, Ian Hennessee, Philip M. Polgreen, Susan E. Beekmann

**Affiliations:** Centers for Disease Control and Prevention, Atlanta, Georgia, USA (K. Benedict, A. Jordan, J.A.W. Gold, D.J. Smith, T. Chiller, I. Hennessee); University of Iowa Carver College of Medicine, Iowa City, Iowa, USA (P.M. Polgreen, S.E. Beekmann)

**Keywords:** cryptococcosis, fungi, HIV/AIDS and other retroviruses, Cryptococcus, diagnosis, HIV, antigen, surveys and questionnaires, United States

## Abstract

We polled infectious disease specialists about cryptococcal antigen screening for patients initiating HIV antiretroviral therapy. Of 215 respondents, 33% reported typically obtaining screening for patients with CD4 counts <200 cells/mm^3^ and 63% for counts <100 CD4 cells/mm^3^. Uncertainty about cryptococcal antigen screening benefits and recommendations suggests opportunities for education and increased screening.

Cryptococcosis is a severe disease caused by the environmental fungus *Cryptococcus*. In the United States, an estimated 3.4–6.5 cases/100,000 population and nearly 5,000 cryptococcosis-associated hospitalizations occur each year; most cases are associated with immunosuppression (*1*,*2*). Approximately one third of patients with cryptococcosis have HIV (*1*,*2*).

Cryptococcal antigen (CrAg) testing is a simple, rapid, inexpensive, and highly accurate diagnostic method. It can detect early asymptomatic cryptococcal infection in blood weeks to months before symptom onset, enabling early treatment and resulting in less illness and death. US federal guidelines recommend routine CrAg screening for persons with HIV and CD4 counts ≤200 cells/mm^3^ (*3*). However, few data exist about CrAg screening practices in the United States. To gain preliminary insights about CrAg screening use and identify potential barriers, we polled members of the Emerging Infections Network (EIN), a provider-based network supported by the Centers for Disease Control and Prevention and the Infectious Disease Society of America (*4*).

EIN emailed a link to an online poll (https://ein.idsociety.org/surveys/survey/181/) 3 times during October 30–November 15, 2024, to its >3,100 network members. We analyzed percentages of responses to questions about CrAg screening utility for patients other than those with advanced HIV; CrAg blood test use for adult and adolescent patients with advanced HIV in 4 clinical scenarios (newly initiating antiretroviral therapy [ART], reinitiating ART, experiencing ART failure, or seriously ill regardless of ART status); barriers to obtaining CrAg testing; and management of patients with a positive CrAg screening test.

In total, 215 EIN members responded. Most were infectious disease physicians who primarily care for adult patients (91%) and practice at university or teaching hospitals (57%) (Table 1). When asked whether patient groups other than those with advanced HIV should routinely receive CrAg screening, 40% of respondents said no, 33% said yes (solid organ or stem cell transplant patients were often mentioned in free-text responses), and 27% were unsure.

**Table 1:** Practice characteristics and testing practices reported by 215 US infectious disease providers in Emerging Infections Network survey of screening for cryptococcal antigenemia, United States, 2024*

Characteristic	Responses, no. (%)†
Primary practice setting	n = 214
Community hospital	48 (22)
Nonuniversity teaching hospital	39 (18)
University hospital	84 (39)
Veterans Affairs or Department of Defense hospital	13 (6)
City, county, or public hospital	10 (5)
Children’s hospital	10 (5)
Cancer facility	2 (1)
Outpatient only	3 (1)
Other	5 (2)
Respondent type	n = 212
Infectious disease physician (primarily for adults)	192 (91)
Infectious disease physician (primarily for children)	14 (7)
Other	3 (1)
Besides adult and adolescent patients with advanced HIV, do you think that other patient groups should be routinely screened for cryptococcal antigenemia?	n = 209
Yes	69 (33)
No	84 (40)
Not sure	56 (27)
Which barriers, if any, concern you or prevent you from obtaining CrAg testing for patients with HIV who are initiating or reinitiating ART and have a CD4 cell count <200 cells/mm^3^?‡	n = 178
Unsure of benefit of CrAg screening	74 (42)
Uncertainty around CrAg screening recommendations	57 (32)
Concern about delaying ART initiation or reinitiation	18 (10)
Long turnaround time for send-out CrAg testing	13 (7)
Difficulty of interpreting CrAg test results	10 (6)
CrAg testing not available at my facility/institution	4 (2)
Challenges with insurance coverage	4 (2)
Other	2 (1)
None of the choices	74 (42)
Which of the following would you consider doing for a patient with a positive CrAg screening test result?‡	n = 181
Not applicable/I do not order CrAg testing	7 (4)
Perform lumbar puncture and order cerebrospinal fluid testing for *Cryptococcus* or CrAg	143 (79)
Evaluate for symptoms of meningitis	140 (77)
Obtain CrAg titer	124 (69)
Treatment with fluconazole while awaiting CSF results	65 (36)
Other	7 (4)
None of these	1 (0.6)

*ART, antiretroviral therapy; CrAg, cryptococcal antigen; CSF, cerebrospinal fluid; N, total number of responses. †Among respondents who answered each question. ‡Respondents could select all that apply.

Of the 215 EIN members who responded, 181 (84%) reported caring for adult or adolescent patients with HIV in the past year. Those respondents participated in the remaining survey questions. The percentages of respondents who reported always or often obtaining CrAg screening for patients with HIV, by CD4 cell count, were as follows: newly initiating ART, <100 cells/mm^3^, 63%; <200 cells/mm^3^, 33%; reinitiating ART, <100 cells/mm^3^, 35%; <200 cells/mm^3^, 17%; experiencing ART failure, <100 cells/mm^3^, 20%; <200 cells/mm^3^, 8%; and seriously ill regardless of ART status, <100 cells/mm^3^, 77%; <200 cells/mm^3^, 68% (Table 2).

**Table 2:** Cryptococcal antigen blood test use for patients with advanced HIV, reported by 215 US infectious disease providers in Emerging Infections Network survey of screening for cryptococcal antigenemia, United States, 2024*

Characteristic	CD4 count, no. (%)†	
	<100 cells/mm^3^	<200 cells/mm^3^
Newly initiating ART	n = 171	n = 166
Never	23 (13)	34 (20)
Rarely	22 (13)	43 (26)
Sometimes	19 (11)	34 (20)
Often	27 (16)	19 (11)
Always	80 (47)	36 (22)
Reinitiating ART	n = 168	n = 160
Never	35 (21)	48 (30)
Rarely	28 (17)	44 (28)
Sometimes	47 (28)	41 (26)
Often	20 (12)	13 (8)
Always	38 (23)	14 (9)
Experiencing ART failure	n = 166	n = 161
Never	44 (27)	57 (35)
Rarely	37 (22)	47 (29)
Sometimes	51 (31)	44 (27)
Often	20 (12)	8 (5)
Always	14 (8)	5 (3)
Seriously ill‡	n = 176	n = 168
Never	8 (5)	11 (7)
Rarely	5 (3)	16 (10)
Sometimes	27 (15)	27 (16)
Often	43 (24)	38 (23)
Always	93 (53)	76 (45)

*ART, antiretroviral therapy. †Among respondents who answered each question. ‡Regardless of ART status.

The primary reported barriers (respondents could choose >1 barrier) to obtaining CrAg screening among patients with HIV and CD4 <200 cells/mm^3^ were uncertainty about the benefit (42%) and uncertainty around CrAg screening recommendations (32%). Ten percent expressed concern about delaying ART, 2% reported CrAg test unavailability, and 42% reported none of the specified barriers. When asked about managing a patient with a positive CrAg screening test result (respondents could choose >1 answer), respondents noted they would perform lumbar puncture and order cerebrospinal fluid testing (79%), evaluate for meningitis symptoms (77%), obtain a CrAg titer (69%), and treat with fluconazole while awaiting cerebrospinal fluid test results (36%).

The poll of EIN members showed moderate (33%–63%, depending on CD4 count) adherence to National Institutes of Health, World Health Organization, and European Confederation of Medical Mycology and International Society for Human & Animal Mycology recommendations for obtaining CrAg screening for patients with advanced HIV who are initiating ART (*3*,*5*,*6*). A modest percentage of respondents (42%) was unsure of the benefit of CrAg screening. In general, limited data exist regarding US CrAg screening implementation (*7*). In addition, approximately 1 in 3 respondents expressed uncertainty about CrAg screening recommendations, and 1 in 10 expressed concerned about delaying ART, which might relate to general awareness of multiple guidelines but unfamiliarity with differences among them. For example, only the World Health Organization (*5*) and the European Confederation of Medical Mycology and International Society for Human & Animal Mycology (*6*) explicitly recommend CrAg screening for patients with HIV who are reinitiating ART, which was not commonly reported (17%–35%) in the poll.

Most respondents (almost 80%) reported that they typically obtain lumbar punctures and evaluate for meningitis symptoms among patients with positive CrAg screening tests, consistent with guidelines. CrAg screening utility for patients without HIV (e.g., transplant recipients) is unclear and has not been well studied (*8*,*9*); 60% of respondents indicated that the practice might be useful or that they were unsure. More research is needed regarding CrAg screening utility among patients without HIV.

A limitation of the survey is that poll respondents might not be generalizable to all US infectious disease physicians. Furthermore, results might overestimate CrAg screening use because of self-selection bias among respondents who chose to participate in the survey based on perceived importance of CrAg screening. Future surveys of CrAg screening practices among other specialist populations might be useful because of increased integration of HIV care into primary care. Our results reveal potential opportunities for improvement in advancing understanding of and adherence to CrAg screening guidelines among a sample of EIN members. 
